# Impact of fasting on thyrotropin and thyroid status during Ramadan in 292 previously well controlled hypothyroid patients. IFTAR study

**DOI:** 10.1007/s12020-022-03242-1

**Published:** 2022-11-07

**Authors:** Tamer Mohamed Elsherbiny

**Affiliations:** Alexandria university, Endocrine division – Alexandria faculty of medicine, Khartoum square, Azarita, Alexandria, Egypt

**Keywords:** Ramadan, Thyrotropin, Fasting, Hypothyroidism

## Abstract

**Purpose:**

Fasting during Ramadan affects thyrotropin both in healthy subjects and hypothyroid patients on adequate levothyroxine replacement. Few studies have addressed this effect in hypothyroid patients with pre-Ramadan euthyroidism. This study aims to report the impact of fasting in a relatively large cohort.

**Methods:**

This was a prospective study including hypothyroid patients who fasted Ramadan during the years 2018, 2019, and 2020 in Alexandria, Egypt. All patients were euthyroid. Patients chosen one of three levothyroxine regimens during Ramadan, regimen 1: 60 min before Iftar, regimen 2: 3–4 h after Iftar, 60 min before Suhor, regimen 3: before the start of next fast, 3–4 h after an early Suhor. Thyroid status was assessed in pre-Ramadan visit and reassessed in post-Ramadan visit within 6 weeks from the end of Ramadan.

**Results:**

The study included 292 hypothyroid patients. Most patients were adherent, 249 patients (85.3%), one sixth of patients were non-adherent, 43 patients (14.7%). Post-Ramadan TSH was 2.13 ± 1.88 mIU/L versus 1.60 ± 0.96 mIU/L pre-Ramadan [*P* = 0.001]. Most patients were still euthyroid post-Ramadan, 233 patients (79.8%), while 59 patients (20.2%) were dysthyroid. Post-Ramadan TSH significantly correlated to pre-Ramadan TSH [*P* < 0.001]. Post-Ramadan TSH was significantly higher in non-adherent patients, 3.57 ± 3.11 mIU/L compared to adherent patients, 1.88 ± 1.44 mIU/L [*P* < 0.001].

**Conclusion:**

Fasting Ramadan in well controlled hypothyroid patients resulted in a significant increase in post-Ramadan TSH, yet 80% the patients remain euthyroid after Ramadan. Post-Ramadan TSH and euthyroidism are related to adherence and pre-Ramadan TSH.

## Introduction

Levothyroxine (L-T4) - being the mainstay treatment for hypothyroidism - has been one of the top ten prescribed medications in the past decade [1]. However, 11% to 42% of hypothyroid patients on L-T4 are under-replaced [2]. Low or non-adherence was reportedly high among L-T4 users, with rates up to 52% in USA and 55% in Lebanon [3, 4]. This lack of adherence to L-T4 is partly related to the fact that it has to be taken daily on an empty stomach, one hour before food and beverages, and avoid interfering drugs for 4 or more hours [5].

Fasting Ramadan entails abstaining from food and drinks from dawn – more than an hour before sunrise – till sundown. In Egypt, years 2018 and 2019, duration of fasting ranged from 15 h:02 min to 15 h:29 min and 14 h:44 min to 15 h:23 min respectively, leaving only 9 h or less for 2 main meals and L-T4 intake for hypothyroid patients [6].

Fasting Ramadan in healthy subjects significantly and gradually increased thyrotropin (TSH) within normal limits – compared to pre-Ramadan values – reaching a maximum at the end of the month and returning back to normal around two months after Ramadan [7]. Fasting Ramadan also flattens TSH diurnal circadian rhythm with decreased midnight and increased afternoon values at the later one third of the month [8].

The aim of the present study is to report the impact of fasting Ramadan on TSH and thyroid status in previously well controlled hypothyroid patients in Alexandria, Egypt.

## Materials and methods

This was a prospective study including Muslim hypothyroid patients willing to fast Ramadan during the years 2018, 2019, and 2020 attending endocrinology outpatient clinic, Alexandria faculty of medicine, Alexandria university, Egypt. All included patients were euthyroid, and stable on the same L-T4 dose for at least 3 months before the start of Ramadan. Exclusion criteria were thyroid cancer patients requiring suppressive therapy, central hypothyroidism, pregnancy, chronic heart failure, liver cirrhosis, renal failure, acute medical or surgical illness at the time of evaluation, to avoid acute and chronic non thyroidal illness syndromes. All patients were explained the nature and aim of the study and signed an informed written consent. The protocol of the study was approved by the ethical committee of Alexandria faculty of medicine, [IRB number 12098].

Patients were free to follow one of three L-T4 regimens during Ramadan, explained in detail previously [6]. In short, regimen 1: to take L-T4 60 min before Iftar and beverages, regimen 2: to take L-T4 3–4 h after Iftar, 60 min before Suhor meal, regimen 3: to take L-T4 before the start of next fast 3–4 h after an early Suhor around midnight. If patients mixed between regimens 1 and 2, this was labeled regimen 4.

Adherence was assessed by interviewing participants during post-Ramadan visit. Non-adherence was defined as stopping food and beverages for less than 3 h before L-T4 tablet(s) or stopping food and beverages for less than 45 min after L-T4 tablet(s). Patients who skipped L-T4 treatment for 2 or more days without making up for their missed doses were excluded from the study.

Thyroid status was assessed for recruited patients in pre-Ramadan visit using TSH. The institution uses electrochemiluminescence immunoassay [ECLIA] on Cobas e 411 (Roche Diagnostics GmbH, Mannheim, Germany). Patients were considered euthyroid when TSH was 0.3–4 mIU/L for patients less than 70 years of age, and 1–5 mIU/L for patients more than 70 years of age according to European thyroid association recommendations [9].

Thyroid status was reassessed in post-Ramadan visit using TSH, provided that this visit comes within 6 weeks from the end of Ramadan. Patients were excluded from the study if post-Ramadan visit was delayed beyond 6 weeks after Ramadan or if they did not report TSH during post-Ramadan visit.

## Statistical methods

Data were fed to the computer and analyzed using IBM SPSS software package version 20.0*.* **(**Armonk, NY: IBM Corp**)**. Qualitative data were described using number and percent. Quantitative data were described using range mean, standard deviation, median (Range). Chi-square test for categorical variables, to compare between different groups. Monte Carlo correction for chi-square when more than 20% of the cells have expected count less than 5. Student’s *t* test for normally distributed quantitative variables, to compare between two studied groups F-test (ANOVA) for normally distributed quantitative variables, to compare between more than two groups. Mann–Whitney test for abnormally distributed quantitative variables, to compare between two studied groups Kruskal–Wallis test for abnormally distributed quantitative variables, to compare between more than two studied groups Wilcoxon signed ranks test for abnormally distributed quantitative variables, to compare between two periods. Spearman coefficient to correlate between two distributed abnormally quantitative variables. Significance of the obtained results was judged at the 5% level.

## Results

### Baseline characteristics

**Fig. 1:**
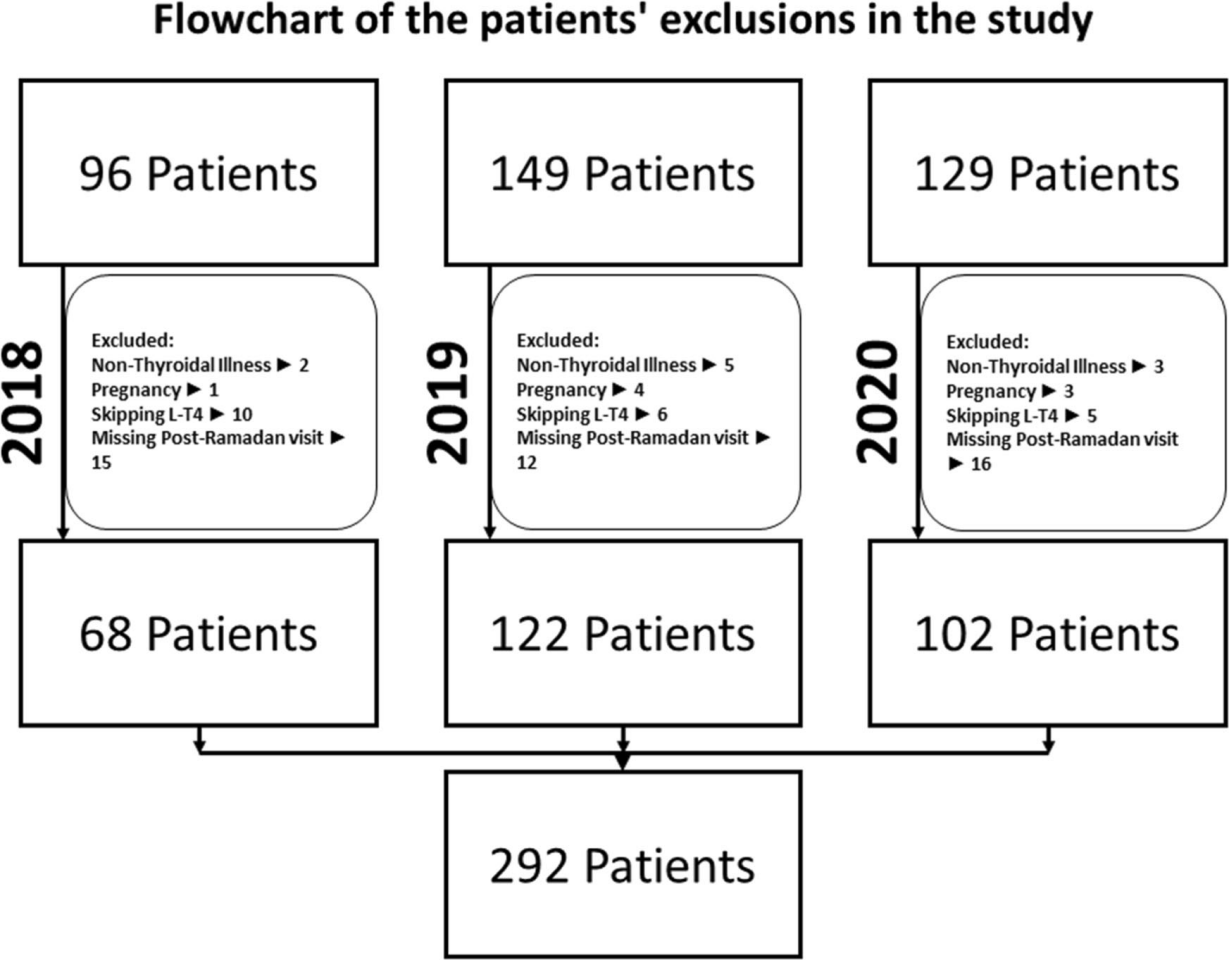
Flow chart of patient’s exclusions in the study

The study included 292 hypothyroid patients, the majority were females [280 patients (95.9%)], and only 12 male patients were included in the study (4.1%). Of the total patients, 68 patients were included in the year 2018 (23.3%), 122 patients in the year 2019 (41.7%), and 102 patients in the year 2020 (35%) [Fig. 1]. Age ranged from 19 to 73 years; and the mean age was 43.5 years. Causes of hypothyroidism included: Hashimoto thyroiditis in 214 patients (73.3%), thyroidectomy in 46 patients (15.8%), unclassified in 21 patients (7.2%), radioiodine ablation in 7 patients (2.4%), and post-partum thyroiditis in 4 patients (1.4%) [Table 1].

**Table 1:** Demographic, clinical data, and thyroid status of studied patients (*n* = 292)

Parameter	No. (%)
Sex	
Female	280 (95.9%)
Male	12 (4.1%)
Age (years)	
<20	3 (1%)
20–<30	39 (13.4%)
30–<40	90 (30.8%)
≥40	160 (54.8%)
Mean ± SD.	43.50 ± 13.25
Median (Min.–Max.)	41 (19–73)
Diagnosis	
HT	214 (73.3%)
Tx	46 (15.8%)
RAI	7 (2.4%)
PPT	4 (1.4%)
Un Classified	21 (7.2%)
Adherence	
Non-adherent	43 (14.7%)
Adherent	249 (85.3%)
L-T4 Regimen	
1	101 (34.6%)
2	127 (43.5%)
3	14 (4.8%)
4	50 (17.1%)
Thyroid status	
Pre	
Euthyroid	292 (100%)
Under-replaced	0 (0%)
Over-replaced	0 (0%)
Post	
Euthyroid	233 (79.8%)
Dysthyroid	59 (20.2%)
Under-replaced	33 (11.3%)
Over-replaced	26 (8.9%)

*HT* Hashimoto thyroiditis, *Tx* thyroidectomy, *RAI* radioiodine ablation, *PPT* post-partum thyroiditis

L-T4 regimen preferences were 101 patients (34.6%), 127 patients (43.5%), 14 patients (4.8%), and 50 patients (17.1%) for regimens 1, 2, 3, and 4 respectively. Most patients were adherent, 249 patients (85.3%), and only one sixth of patients were non-adherent, 43 patients (14.7%) [Table 1].

**Fig. 2:**
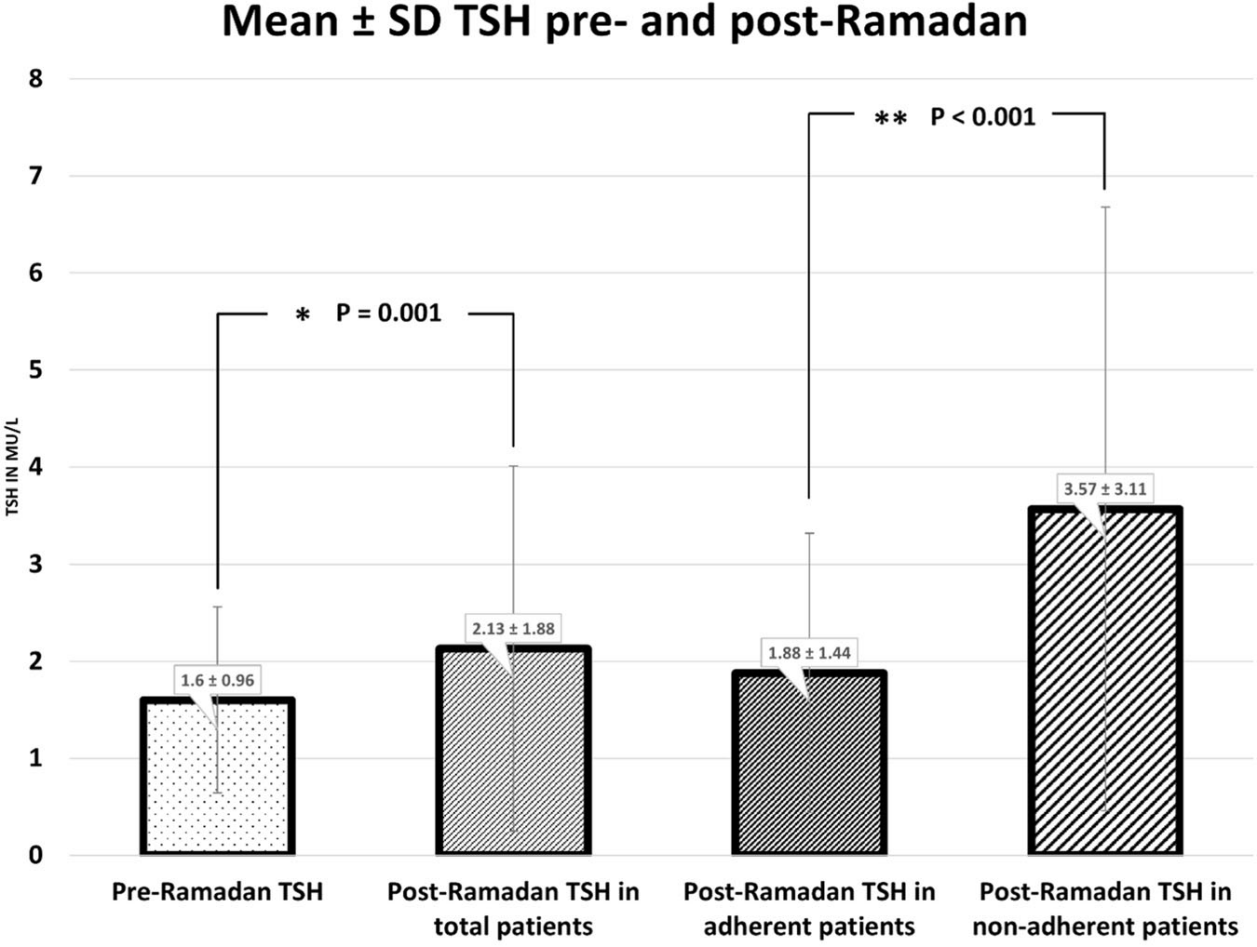
Mean pre-Ramadan TSH, post-Ramadan TSH in the total patients, adherent, and non-adherent patients. *: Statistically significant at *p* ≤ 0.05, *p* value for comparing between pre- and post-Ramadan TSH using Wilcoxon signed ranks test. **: Statistically significant at *p* ≤ 0.05, *p* value for comparing between adherent and non-adherent patients using Mann–Whitney test

Fasting Ramadan significantly increased post-Ramadan TSH relative to pre-Ramadan TSH. Post-Ramadan TSH was 2.13 ± 1.88 mIU/L versus 1.60 ± 0.96 mIU/L pre-Ramadan [*P* = 0.001]. Post-Ramadan TSH was significantly higher in non-adherent patients, 3.57 ± 3.11 mIU/L compared to adherent patients, 1.88 ± 1.44 mIU/L [*P* < 0.001] [Fig. 2].

Most patients were still euthyroid post-Ramadan, 233 patients (79.8%), while 59 patients (20.2%) were dysthyroid in the post Ramadan visit [Table 1]. Adherence rates were significantly higher in patients with post-Ramadan euthyroidism (89.3%) versus only (69.5%) in patients with post-Ramadan dysthyroidism [*P* < 0.001].

To show the effect of age on post-Ramadan TSH, patients were classified into 5 age groups as shown in Table 2. Post-Ramadan TSH increased significantly only in the 2 younger age groups, less than 30 years, and 30 to <40 years [Table 2]. Rates of adherence increased with increasing age, with the lowest adherence in patients < 30 years of age 33/42 (78.6%), intermediate adherence in patients from 30 to <60 years 175/205 (85.4%), and highest adherence in patients 60 years or older 41/45 (91.1%).

**Table 2:** Comparison between pre-Ramadan and post-Ramadan TSH according to age, etiology of hypothyroidism, and L-T4 regimen preference

	TSH	*N*	Pre-Ramadan TSH	Post-Ramadan TSH	Z	*p*
Total	Mean ± SD.	292	1.60 ± 0.96	2.13 ± 1.88	3.199^*^	0.001^*^
	Median (Min.–Max.)		1.4 (0.3–4.80)	1.68 (0.01–15.40)		
Age (years)	<30					
	Mean ± SD.	42	1.55 ± 0.91	2.56 ± 2.14	2.313^*^	0.021^*^
	30–<40					
	Mean ± SD.	90	1.61 ± 0.99	2.41 ± 2.34	2.386^*^	0.017^*^
	>40–<50					
	Mean ± SD.	51	1.52 ± 0.88	2.03 ± 1.41	1.711	0.087
	50–<60					
	Mean ± SD.	64	1.46 ± 0.81	1.70 ± 1.36	0.853	0.394
	≥60					
	Mean ± SD.	45	1.93 ± 1.18	1.91 ± 1.54	0.587	0.577
Diagnosis	HT					
	Mean ± SD.	214	1.60 ± 0.95	2.10 ± 1.73	2.673^*^	0.008^*^
	Tx					
	Mean ± SD.	46	1.48 ± 0.97	1.95 ± 2.54	0.415	0.678
	RAI					
	Mean ± SD.	7	1.23 ± 1.27	2.32 ± 2.11	1.352	0.176
	PPT					
	Mean ± SD.	4	1.64 ± 0.93	3.77 ± 1.63	1.461	0.144
	Un Classified					
	Mean ± SD.	21	2.06 ± 0.88	2.46 ± 1.52	1.321	0.187
Regimen number	1					
	Mean ± SD.	101	1.57 ± 0.95	2.08 ± 1.70	1.834	0.067
	2					
	Mean ± SD.	127	1.57 ± 0.94	2.24 ± 2.14	2.563^*^	0.010^*^
	3					
	Mean ± SD.	14	2.16 ± 1.24	1.80 ± 1.34	0.597	0.551
	4					
	Mean ± SD.	50	1.60 ± 0.93	2.06 ± 1.63	1.347	0.178

*HT* Hashimoto thyroiditis, *Tx* thyroidectomy, *RAI* radioiodine ablation, *PPT* post-partum thyroiditis, *Z* Wilcoxon signed ranks test, *p* p value for comparing between pre and post

*Statistically significant at *p* ≤ 0.05

Also, patients were classified according to the etiology of hypothyroidism, post-Ramadan TSH was significantly raised only in those who have Hashimoto thyroiditis. When patients were classified according to their preferred L-T4 regimen, post-Ramadan TSH was significantly raised only in patients who followed regimen 2. [Table 2]

Post-Ramadan TSH significantly correlated to pre-Ramadan TSH (Spearman coefficient 0.268 [*P* < 0.001]), and age (Spearman coefficient –0.117 [*P* = 0.046]).

## Discussion

The present study showed that in previously well controlled hypothyroid patients, fasting Ramadan was associated with a significant increase in TSH from 1.60 ± 0.96 mIU/L to 2.13 ± 1.88 mIU/L [*P* = 0.001]. Almost 80% of patients were still euthyroid post-Ramadan. 85.3% of the patients were adherent to L-T4 regimen of their choice. Non-adherence was associated with significantly higher TSH, whereas adherent patients constituted almost 90% of patients who maintained euthyroidism in post-Ramadan. Pre-Ramadan TSH and age were significantly associated with post-Ramadan TSH.

Four published studies reported the impact of fasting Ramadan in previously well controlled hypothyroid patients with numbers of included patients ranging from 47 patients by Karoli et al. in 2013 to 112 patients by El-Kaissi et al. in 2020. The present study included 292 well controlled hypothyroid patients [10–13].

In healthy subjects fasting Ramadan, TSH increases significantly within normal reference range, and returns to pre-Ramadan values two months after Ramadan [7]. In adequately replaced hypothyroid patients, post-Ramadan TSH increased significantly compared to pre-Ramadan TSH in 2 studies by Dabbous et al. and El-Kaissi et al. whereas more than 60% of patients showed an increase in TSH without statistical significance in the other 2 studies by Karoli et al. and Dellal et al. [10–13]. El-Kaissi et al. reported that 32% of their patients were dysthyroid after Ramadan, whereas only 20.2% of the patients in the present study were dysthyroid [13].

Post-Ramadan TSH was negatively correlated with age (Spearman coefficient –0.117 [*P* = 0.046]), meaning that older age was associated with lower post-Ramadan TSH. Also, post-Ramadan TSH increased significantly only in patients less than 40 years of age, whereas older patients did not show a significant increase. An opposite finding was reported by El-Kaissi et al. who found that older patients had a higher post-Ramadan TSH [13]. Current findings may be explained by a better adherence to L-T4 with increasing age, recently reported in a middle eastern country [14]. When patients were classified by age, the highest adherence rate was found in patients 60 years or older (91.1%), whereas the lowest adherence rate was found in patients younger than 30 years (78.6%).

Only hypothyroid patients due to Hashimoto thyroiditis showed a significant increase in post-Ramadan TSH. Hashimoto thyroiditis accounted for 73.3% of the recruited patients. Number of patients attributed to other causes may have been too small to show statistical significance. Also, only patients following L-T4 regimen 2 showed a significant increase in post-Ramadan TSH.

Levothyroxine intake regimens offered to our patients are based on American thyroid association recommendations as explained before [15]. L-T4 regimens offered to patients in previous studies included: with iftar, 30 min before iftar or 30 min before suhor, or 2 h after iftar or food [10–13]. These regimens are not consistent with ATA recommendations to optimize absorption; although, it was considered convenient by most patients in one study by Dabbous et al. however, the authors of this study considered these time intervals insufficient for optimal absorption due to the nature of foods commonly consumed during Ramadan [11].

85% of the patients in the present study were adherent to L-T4 regimen of their choice. Reported rates of adherence range from 25% reported by Karoli et al., 35–42% by Dabbous et al. and up to 75% reported by Sheikh et al. [10, 11, 16]. 82% adherence was previously reported by our institution following the same L-T4 regimens used in the present study [6]. Having multiple L-T4 regimen and freedom of choice of the regimen that suits every patient’s priority may have contributed to this high rate of adherence.

Non-adherence resulted in significantly higher TSH compared to adherence whereas 89% of patients who maintained euthyroidism post-Ramadan were adherent. Karoli et al. found that meal-levothyroxine interval correlated negatively to post-Ramadan TSH [10].

With an estimated number of about 48 million Muslims with hypothyroidism requiring L-T4 replacement and practicing Ramadan fasting yearly, the currently available literature addressing the impact of fasting Ramadan on thyroid status is quite limited and unsatisfactory compared to the literature for example covering the topic of Ramadan fasting and diabetes [6, 17]. Future research is needed to report experience with fasting Ramadan in different locations and different times of the year, and to answer research questions like the need to raise L-T4 dose temporarily during Ramadan and the utility of weekly or biweekly L-T4 during Ramadan fasting.

The present study has several strengths. The only study to be conducted in 3 consecutive Ramadan months, in 3 consecutive years allowing the inclusion of a large number of patients. The largest number of recruited well controlled hypothyroid patients fasting during Ramadan in a single study. In fact, the number of included patients – 292 patients – is almost equivalent to the numbers of included patients in the 4 similarly designed studies combined, 317 patients [10–13]. The only study to offer L-T4 regimens in accordance with the latest American thyroid association recommendations for optimal L-T4 absorption, as opposed to regimens in previous studies with time intervals that may cause a suboptimal absorption, which may explain the highest rate of post-Ramadan euthyroidism (79.8%) versus previously reported (68%) [13]. The only study to be conducted in a north African country – Egypt – as opposed to other studies conducted in countries from west Asia in Turkey to south Asia in India, thus, our patients were of different ethnicities, social and dietary habits.

A major limitation for this study and others dealing with fasting Ramadan is the fact that duration of fasting and atmospheric temperatures differ from one country to another according to latitude and for the same country from year to year as Ramadan moves from one season to another every 8 years, meaning that results can’t be generalized for all countries or even for the same country in different seasons of the year.

In conclusion, – based on the present data from 292 hypothyroid patients – fasting Ramadan in well controlled hypothyroid patients resulted in a significant increase in post-Ramadan TSH, yet 80% the patients remain euthyroid after Ramadan. Post-Ramadan TSH and euthyroidism are related to adherence, L-T4 regimen preference, age, and pre-Ramadan TSH.
